# Modulation of Gap Junction Coupling Within the Islet of Langerhans During the Development of Type 1 Diabetes

**DOI:** 10.3389/fphys.2022.913611

**Published:** 2022-06-28

**Authors:** Nikki L. Farnsworth, Robert A. Piscopio, Wolfgang E. Schleicher, David G. Ramirez, Jose G. Miranda, Richard K. P. Benninger

**Affiliations:** ^1^ Department of Chemical and Biological Engineering, Colorado School of Mines, Golden, CO, United States; ^2^ Barbara Davis Center for Diabetes, Universty of Colorado Anschutz Medical Campus, Aurora, CO, United States; ^3^ Department of Bioengineering, University of Colorado Anschutz Medical Campus, Aurora, CO, United States

**Keywords:** calcium signaling, connexin36, cytokines, islet dysfunction, NOD mouse, type 1 diabetes

## Abstract

In type 1 diabetes (T1D), islet dysfunction occurs prior to diabetes onset. Pro-inflammatory cytokines can disrupt insulin secretion and Ca^2+^ homeostasis. Connexin36 (Cx36) gap junctions electrically couple β-cells to coordinate glucose-stimulated Ca^2+^ and insulin secretion. Cx36 gap junction coupling can also protect against cytokine-induced apoptosis. Our goal was to determine how islet gap junction coupling and Ca^2+^ dynamics are altered in mouse models of T1D prior to diabetes. Glucose tolerance was assessed in NOD and immunodeficient NOD-RAG1KO mice at 6–12 weeks age. Glucose-stimulated insulin secretion, Ca^2+^ dynamics, and gap junction coupling were measured in islets isolated at each age. Gap junction coupling was also measured in islets from mice that underwent transfer of diabetogenic splenocytes and from chromograninA knockout NOD mice. Cell death was measured in islets isolated from wild-type, Cx36 knockout or Cx36 over-expression mice, each treated with a cocktail of pro-inflammatory cytokines and K_ATP_ or SERCA activators/inhibitors. NOD mice over-expressing Cx36 were also monitored for diabetes development, and islets assessed for insulitis and apoptosis. NOD and NOD-RAG1KO controls showed similar glucose tolerance at all ages. Ca^2+^ dynamics and gap junction coupling were disrupted in islets of NOD mice at 9 weeks, compared to controls. Transfer of diabetogenic splenocytes also decreased gap junction coupling. Islets from chromograninA knockout mice displayed normal coupling. Overexpression of Cx36 protected islets from cytokine-induced apoptosis. A knockout of Cx36 amplified cytokine-induced apoptosis, which was reversed by K_ATP_ activation or SERCA activation. Cx36 overexpression in NOD mice delayed diabetes development compared to NOD controls. However, apoptosis and insulitis were not improved. Decreases in islet gap junction coupling occur prior to T1D onset. Such decreases alter islet susceptibility to apoptosis due to altered Ca^2+^. Future studies will determine if increasing Cx36 gap junction coupling in combination with restoring Ca^2+^ homeostasis protects against islet decline in T1D.

## Introduction

Type 1 diabetes (T1D) is characterized by the progressive immune destruction of insulin producing β-cells in the pancreatic islets of Langerhans. Activated immune cells in the pancreas initiate β-cell death in part through secretion of high levels of Th1 cytokines, including tumor necrosis factor-α (TNF-α) from activated macrophages and interleukin-1β (IL-1β) and interferon-γ (IFN-γ) from activated T-cells (Cnop, Welch et al., 2005; Padgett, Broniowska et al., 2013). Early in the progression of T1D, disruptions to insulin secretion have been reported prior to loss of glucose homeostasis, indicating a potential role for islet dysfunction in the pathogenesis of T1D (Lo, Hawa et al., 1992; Ize-Ludlow, Lightfoot et al., 2011; Soleimanpour and Stoffers 2013). High levels of pro-inflammatory cytokines can also increase β-cell endoplasmic reticulum (ER) and oxidative stress early in T1D progression (Lu, Liu et al., 2020). *In vitro*, pro-inflammatory cytokines reduce insulin secretion (Wang, Guan et al., 2010) and alter intracellular free-calcium (Ca^2+^) and ER calcium dynamics (Ramadan, Steiner et al., 2011; Kono, Ahn et al., 2012). Our previous work demonstrated *in vitro*, that cytokines decreased connexin36 (Cx36) gap junction coupling and disrupted the coordination of Ca^2+^ dynamics (Farnsworth, Walter et al., 2016). However, changes in islet Cx36 gap junction coupling and Ca^2+^ signaling dynamics have not been studied in T1D.

Cx36 gap junctions form channels between β-cells that mediates electrical coupling and regulates islet function in a glucose-dependent manner (Serre-Beinier, Gurun et al., 2000; Serre-Beinier, Bosco et al., 2009). At high glucose, Cx36 gap junction coupling coordinates glucose-induced membrane depolarization, pulsatile intracellular Ca^2+^ signaling, and insulin secretion across the islet (Ravier, Guldenagel et al., 2005; Benninger, Zhang et al., 2008; Farnsworth and Benninger 2014). At low glucose, Cx36 gap junction coupling coordinates hyper-polarizing electrical currents across the islet to suppress intracellular Ca^2+^ and insulin secretion (Benninger, Head et al., 2011; Farnsworth and Benninger 2014). Loss of Cx36 gap junction coupling between β-cells results in a loss of first phase insulin secretion and reduced glucose tolerance in mice (Head, Orseth et al., 2012; Perez-Armendariz 2013), similar to what is observed in pre-diabetic individuals (Lin, Hsia et al., 2010; Cersosimo, Triplitt et al., 2018). While decreases in first-phase insulin secretion are also a hallmark of preclinical T1D (Daneman 2006; Sosenko, Skyler et al., 2013), it is unknown whether changes in Cx36 gap junction coupling and Ca^2+^ signaling dynamics also occur early in the progression of T1D, when islet dysfunction is observed.

The goal of this study was to determine if changes in islet Cx36 gap junction coupling and Ca^2+^ signaling dynamics occur early in the progression of T1D, prior to changes in glucose tolerance. We utilized the NOD mouse model of T1D to study changes in Cx36 prior to disease onset, as progression in this mouse model is similar to human disease (Pearson, Wong et al., 2016). In addition to regulating islet intracellular Ca^2+^ signaling dynamics, Cx36 gap junction coupling also protects against β-cell apoptosis (Klee, Allagnat et al., 2011; Allagnat, Klee et al., 2013; Cigliola, Populaire et al., 2016). Specifically, Cx36 protects against cytokine-induced β-cell death *in vitro via* downregulation of oxidative and ER stress (Calabrese, Zhang et al., 2003; Ravier, Guldenagel et al., 2005; Allagnat, Klee et al., 2013). *In vivo*, overexpression of Cx36 resulting in increased β-cell coupling protects against streptozotocin (STZ) induced β-cell death (Klee, Allagnat et al., 2011). Therefore, this study also sought to determine if modulation of Cx36 and intracellular Ca^2+^ could protect against β-cell death and delay the onset of diabetes in the NOD mouse model.

## Research Design and Methods

### Animal Care

All experiments utilizing mice were approved by the University of Colorado Denver Institutional Animal Care and Use Committee [Protocol # B-95817(05)1D]. Mice were housed in a temperature-controlled facility with access to food and water *ad libitum* on a 12 h light/dark cycle. In NOD mice diabetes progression was monitored by weekly ad lib glucose measurements taken from the tail vain. Mice with a blood glucose level >250 mg/dl for three consecutive measurements were considered diabetic and were euthanized *via* CO_2_ and cervical dislocation to minimize distress. All animals were bred in house except where otherwise noted.

### Generation of Transgenic Mice

Cx36 knockout (KO) mice were generated as previously described (Degen, Meier et al., 2004). The rat insulin promoter driven Cx36 (RIPCx36) plasmid construct was a gift from Dr. Philippe Klee (Klee, Allagnat et al., 2011). The expression cassette was removed by enzymatic digestion, purified, and injected into C57BL/6 embryos at the University of Colorado Anschutz Medical Campus Transgenic and Gene Targeting Core. Mice carrying the transgene were identified by PCR using primers designed for the mutant Cx36. RIPCx36 mice were backcrossed onto the NOD background by identifying two transgenic founders and successive breeding with NOD mice from a colony maintained at the University of Colorado Anschutz Medical Campus for up to 10 generations. Mice from generations 1–10 were used to characterize the overexpression of Cx36. Mice from generation 2 were backcrossed onto the NOD background using a “speed congenic” technique. Within 4 generations of backcrossing, >99% NOD genetic background was achieved. Mice from generations 5–10 were monitored to determine the diabetic phenotype of the colony compared to the phenotype of the original NOD colony. Littermate mice negative for RIPCx36 served as controls (WT-NOD).

### Fasting Glucose Tolerance Test

Mice were fasted with access to water ad lib for 16 h prior to performing the GTT. Fasting glucose was measured *via* the tail vein and 200 mg/kg glucose was given by intraperitoneal (IP) injection. Blood glucose was monitored over 2 h post glucose bolus.

### Islet Isolation and Culture

Pancreata were isolated from female 6, 9, or 12-week old NOD and NOD-RAG1KO mice; male and female 12-week old WT-NOD, RIPCx36-NOD; or female 12-week old chromogranin A knockout NOD (ChGAKO-NOD) mice. Female NOD mice were selected for experiments investigating diabetes onset, as they more reliably progress to diabetes than their male counterparts (Matthews, Xue et al., 2015). Male and female RIPCx36-NOD mice were selected to determine differences in Cx36 gene expression in the presence and absence of diabetes. Briefly, animals were injected with 100 mg/kg ketamine and 8 mg/kg xylazine and euthanized *via* exsanguination prior to inflating the pancreas with a collagenase solution and removing the pancreas, per our approved animal protocol. Islets were isolated by enzymatic digest of the pancreas with 2.5 mg/ml collagenase (C9263, Millipore-Sigma, Saint Louis, MO) at 37°C. Islets were handpicked into 1640 RPMI Medium (Millipore-Sigma) with 10% FBS, 10,000 U/mL Penicillin and 10,000 μg/ml Streptomycin and incubated overnight at 37°C and 5% CO_2_. Islet from WT, Cx36 KO, and RIP Cx36 mice were also cultured with a cytokine cocktail containing 10 ng/ml tumor necrosis factor-α (TNF-α, 410-MT-010/CF, R&D Systems, ED_50_ = 8–50 pg/ml), 5 ng/ml interleukin-1 β (IL-1β, 401-ML-005/CF, R&D systems, ED_50_ = 2–10 pg/ml), and 100 ng/ml interferon-γ (IFN-γ, 485-MI-100/CF, R&D Systems, ED_50_ = 0.3–0.9 ng/ml) for 24 h in culture media.

### Adoptive Transfer

Splenocytes were isolated from female NOD mice with ad lib blood glucose levels >250 mg/dl by manual dissociation in cold Hanks Balanced Salt Solution (HBSS). Leukocytes were counted and one dose of 20 × 10^6^ leukocytes in HBSS was injected into each 12–14-week old NOD-scid (Jackson Laboratory, Bar Harbor, ME) adoptive transfer recipient. NOD-scid vehicle controls were injected with an equal volume of HBSS. Islets were isolated as previously described 2 weeks after adoptive transfer.

### Intracellular Calcium Imaging and Analysis

Isolated islets were incubated with 4 µM Fluo-4 AM (Thermo Fisher Scientific, Waltham, MA) in BMHH buffer containing 125 mM NaCl, 5.7 mM KCl, 2.5 mM CaCl_2_, 1.2 mM MgCl_2_, 10 mM HEPES, 0.1% bovine serum albumin (BSA), and 2 mM glucose at room temperature for 2 h in the dark. Islets were transferred to MatTek glass-bottom imaging dishes (MatTek, Ashland, MA) with fresh BMHH with either 2 mM or 5 mM glucose prior to imaging. Islets were imaged on one of three microscopes: 1) a Nikon Eclipse-Ti wide field microscope (Nikon, Melville, NY) with a x20 0.75 NA Plan Apo objective, a 490/40 nm band-pass excitation filter and a 525/36 nm band-pass emission filter, 2) a Zeiss 780 laser scanning microscope with a x40 1.2 NA water c-apochromat objective using a 488 nm Ar^+^ laser line and a QUASAR spectral detector, 3) a Zeiss 800 laser scanning microscope with a x40 1.2 NA c-apochromat objective using a 488 nm Ar^+^ laser with a 525/20 nm emission filter. Islets were imaged at 37°C, where images were acquired every second for 2.5–5 min following incubation at 2 mM glucose for 2 h, 5 mM glucose for 2 h, 11 mM glucose for 10 min, or 20 mM glucose for 10 min. The fraction of islet area active at 2 mM or 5 mM glucose and the fraction of islet area synchronized at 11 mM or 20 mM glucose was determined as previously described (Hraha, Bernard et al., 2014) using MATLAB (Mathworks, Natick, MA).

### Fluorescence Recovery After Photobleaching Measurement of Connexin36 Gap Junction Coupling

FRAP measurement of Cx36 coupling was performed as described in Farnsworth et al. (2014). Immediately after isolation, islets were adhered to MatTek glass bottom dishes with Cell-Tak (Thermo Fisher Scientific) per the manufacturer’s instructions and cultured overnight in RPMI as previously described. Prior to imaging, islets were incubated with 12.5 µM rhodamine 123 in Hanks Balanced Salt Solution (HBSS) for 30 min at 37°C. Islets were washed with fresh HBSS and imaged on either a Zeiss 510 LSM with a 488 nm Ar^+^ laser, a 700/488 nm short pass dichroic beam splitter, a 490 nm long pass secondary beam splitter, and a 505 nm long pass emission filter or a Zeiss 800 LSM as previously described for Ca^2+^ imaging. As described in Farnsworth et al. (2014), half of each islet was photobleached and the fluorescence recovery in the bleached region was measured over time. The fluorescence recovery rate, which correlates with Cx36 gap junction coupling, was calculated from the reverse exponential fluorescence recovery curve.

### Calcium Modulation Treatments and Cell Death Measurement

Islets from WT or Cx36 KO mice were cultured for 24 h with and without the cytokine cocktail mentioned previously. Islets were also cultured for 24 h with and without: 250 µM of the K_ATP_ channel activator Diazoxide (Millipore-Sigma), 20 mM of the K channel inhibitor tetraethylammonium (TEA, Millipore-Sigma), 1 µM of the sarco/endoplasmic reticulum Ca^2+^ ATPase (SERCA) activator Ochratoxin A (Millipore-Sigma), and 1 µM of the SERCA inhibitor thapsigargin (Millipore). After incubation, islets were stained with 0.5 mg/ml fluorescein diacetate (Sigma) and 0.1 mg/ml propidium iodide (Sigma) in PBS to identify live and dead cells respectively. Islets were imaged on a Zeiss 800 scanning laser confocal microscope where 3 optical sections per islets were obtained and quantified manually for live and dead cells using ImageJ for 5–10 islets per condition.

### Connexin36 Gene Expression

Islets were isolated from 8 to 12-week old male and female NOD or RIPCx36-NOD littermates as previously described. Islet mRNA was isolated using the RNeasy mini kit (Qiagen, Germantown, MD) per the manufacturer’s instructions and RNA was eluted in 30 µl DI water. RNA was converted to cDNA using the 1st Strand cDNA synthesis kit (OriGene, Rockville, MD) per the manufacturer’s instructions with 1 µg of RNA per reaction. Primers for Cx36 were purchased from OriGene (NM_010290, forward primer: GTGGTGCTCAATCTGGCTGAAC, reverse primer: GACTGAGTCCTGCCGAAATTGG) and primers for the housekeeping gene hypoxanthine phosphoribosyltransferase 1 (HPRT1) are as follows: 5′-TGGATACAGGCCAGACTTTGTT-3′ and 3′-GGGAAAATACAGCCAACACTGC-5′. PCR reactions were created using Eppendorf Matercycler Gradient Thermocycer and run on a CFX96 Real-Time System Thermocycler real-time PCR machine with Pre-soak 95°C for 10 min/Denaturation 95°C for 15 s/Annealing 60°C for 30 s for 40 cycles cycle/temp/time. Cycle time (Ct) values were calculated using Bio-Rad CFX Manager Software software and the relative change in Cx36 gene expression was calculated using the ΔΔCt method, where wild type (WT) NOD islets are the control samples (Ct_control_) and RIPCx36-NOD islets are the experimental samples (Ct_exp_). Relative changes in gene expression were calculated as follows:

1) ΔCt_control_ = Ct_WT-NOD_—Ct_HPRT1_
2) ΔCt_exp_ = Ct_RIPCx36-NOD_—Ct_HPRT1_
3) ΔΔCt = 2^-(ΔCtexp-AVG(ΔCtcontrol))^

### Immunohistochemistry and Insulitis Scoring

Pancreata from 12-week old NOD and RIPCx36-NOD female mice were isolated and fixed in 4% paraformaldehyde at 4°C overnight. Fixed pancreata were embedded in paraffin and sliced into 5 µm sections. Sections were imaged on a Nikon Eclipse Ti widefield microscope (Nikon, Melville, NY) with ANDOR color camera with a x40 objective. Insulitis was determined from pancreatic sections stained with hematoxylin and eosin. Islets with no insulitis were given a score of 0, islets with peri-insulitis were given a score of 1, islets with <50% infiltrated area were given a score of 2, and islets with >50% infiltrated area were given a score of 3. The insulitis scores were averaged for all islets on 6 pancreatic sections taken from different regions of the pancreas (∼100 µm apart).

Subsequent pancreas sections were deparaffinized, permeabilized with 0.25% Triton-X-100 (Sigma) and stained for TUNEL positive cells with the ClickiT Plus TUNEL Assay (ThermoFisher) per the manufacturer’s instructions. TUNEL staining was verified with DNase treated pancreatic tissue slices. Slides were then stained with guinea pig anti-insulin primary antibody (ab7842, Abcam) diluted 1:100 in PBS with 5% normal donkey serum (NDS) at 4°C overnight. Slides were rinsed in PBS, then stained with AlexaFluor 647 Donkey anti-guinea pig secondary antibody (706-605-148, Jackson Immuno Research) diluted 1:500 in PBS with 5% NDS at room temperature for 2 h. Slides were rinsed, then mounted in Fluoromount with DAPI (ThermoFisher) and sealed. Insulin antibody staining was verified on pancreatic tissue slices from NOD-RAG1KO mice. Slides were imaged on a Nikon Eclipse Ti widefield microscope with x20 0.75 NA Plan Apo objective. DAPI was imaged with a 402/50 nm band-pass excitation filter and a 455/50 nm band-pass emission filter, TUNEL positive cells labeled with AlexaFluor488 were imaged with a 490/20 nm band-pass excitation filter and a 525/36 nm band-pass emission filter, and AlexaFluor647 was imaged with a 555/25 nm band-pass excitation filter and a 605/52 band-pass emission filter. Images of the whole pancreas slice were obtained using a tile scan and image stitching with 15% image overlap. TUNEL positive and insulin positive cells were quantified using ImageJ and insulin positive pancreas area was determined using MATLAB (MathWorks, Natick, MA).

### Statistical Analysis

To determine statistical significance, analysis of variance (ANOVA) with Tukey’s post hoc analysis or a student’s t-test was used where indicated. In each figure panel, all genotypes and treatments are compared against one another; however, only differences that were found to be statistically significant with a *p*-value <0.05 were reported in all figures. To determine clustering of NOD coupling and Ca^2+^ data, k-means clustering was used in combination with a t-test to determine significance between identified clusters of data. To determine statistical differences in survival of the WT NOD and RIPCx36-NOD mouse colonies, Kaplan-Meier survival analysis with a Breslow test was used where indicated.

## Results

### NOD Mice are Normoglycemic at 12 weeks of Age

**FIGURE 1:**
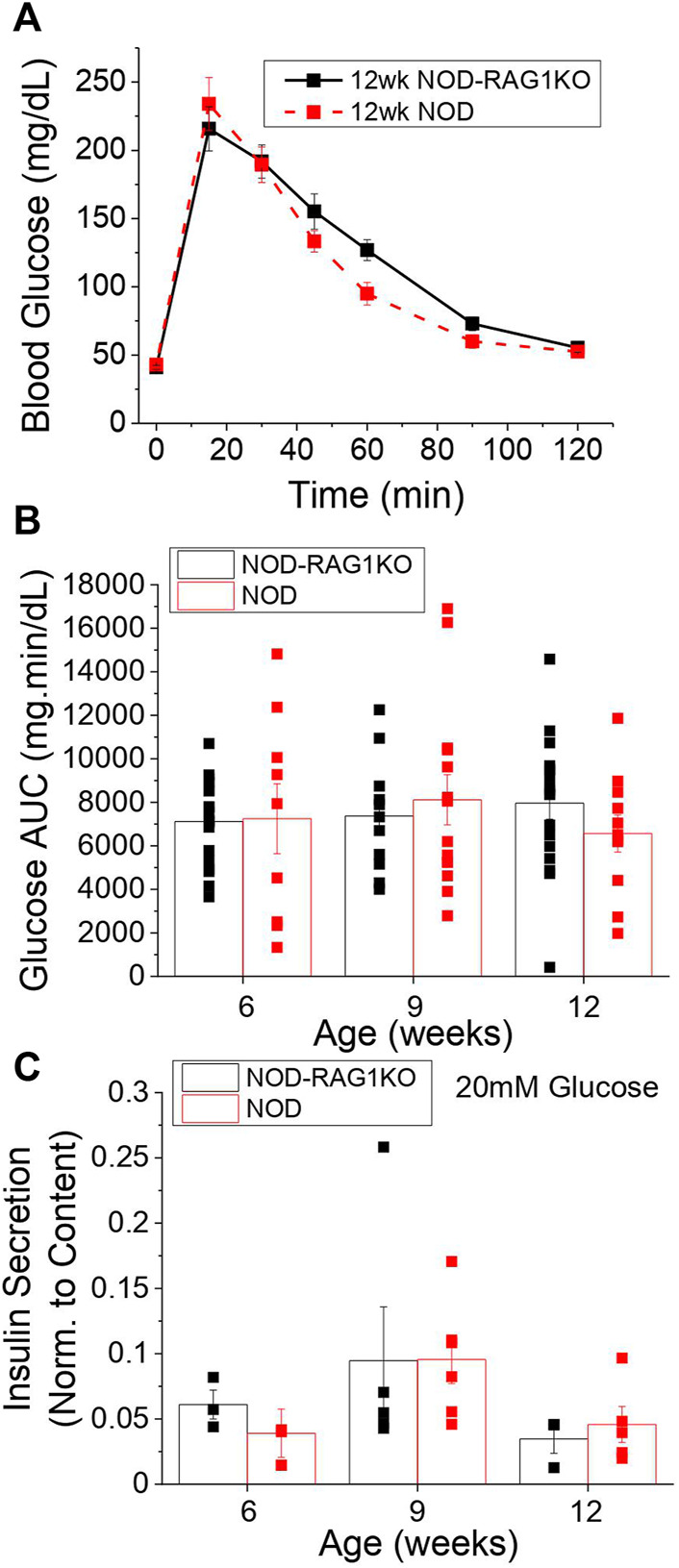
NOD mice are normoglycemic at 12 weeks of age. **(A)** Blood glucose levels in 12-week old NOD (*n* = 7–13) and NOD-RAG1KO (*n* = 8–15) mice following a glucose tolerance test starting at time 0. **(B)** Glucose area under the curve for 6, 9, and 12 week old NOD (red) and NOD-RAG1KO (black) mice calculated from blood glucose levels measured over 2 h following glucose challenge in as A. **(C)** Insulin secretion normalized to insulin content in isolated islets from 6, 9, and 12 week old NOD (*n* = 4–6) and NOD-RAG1KO (*n* = 3–5) mice at 20 mM glucose. Data in all panels represents the mean ± SEM. In B and C, data from individual mice are represented by black and red squares for NOD-RAG1KO and NOD mice respectively. All animals are female unless otherwise noted.

To determine the optimal time frame to study pre-symptomatic diabetes in the NOD mouse we assessed glucose homeostasis in female mice at 6, 9, and 12 weeks *via* GTT. No significant changes in glucose tolerance test (Figure 1A, Supplementary Figures S1A,B) or area-under-the-curve (AUC) from the GTT curves (Figure 1B) were found at 6, 9, or 12 weeks compared to age-matched NOD-RAG1KO controls. Islets isolated from NOD and NOD-RAG1KO mice secreted similar amounts of insulin at 2 mM (Supplementary Figure S1C) and 20 mM glucose (Figure 1C) and had similar insulin content (Supplementary Figure S1D) at all ages investigated.

### Islets From Normoglycemic NOD Mice Show Disrupted Calcium Signaling

**FIGURE 2:**
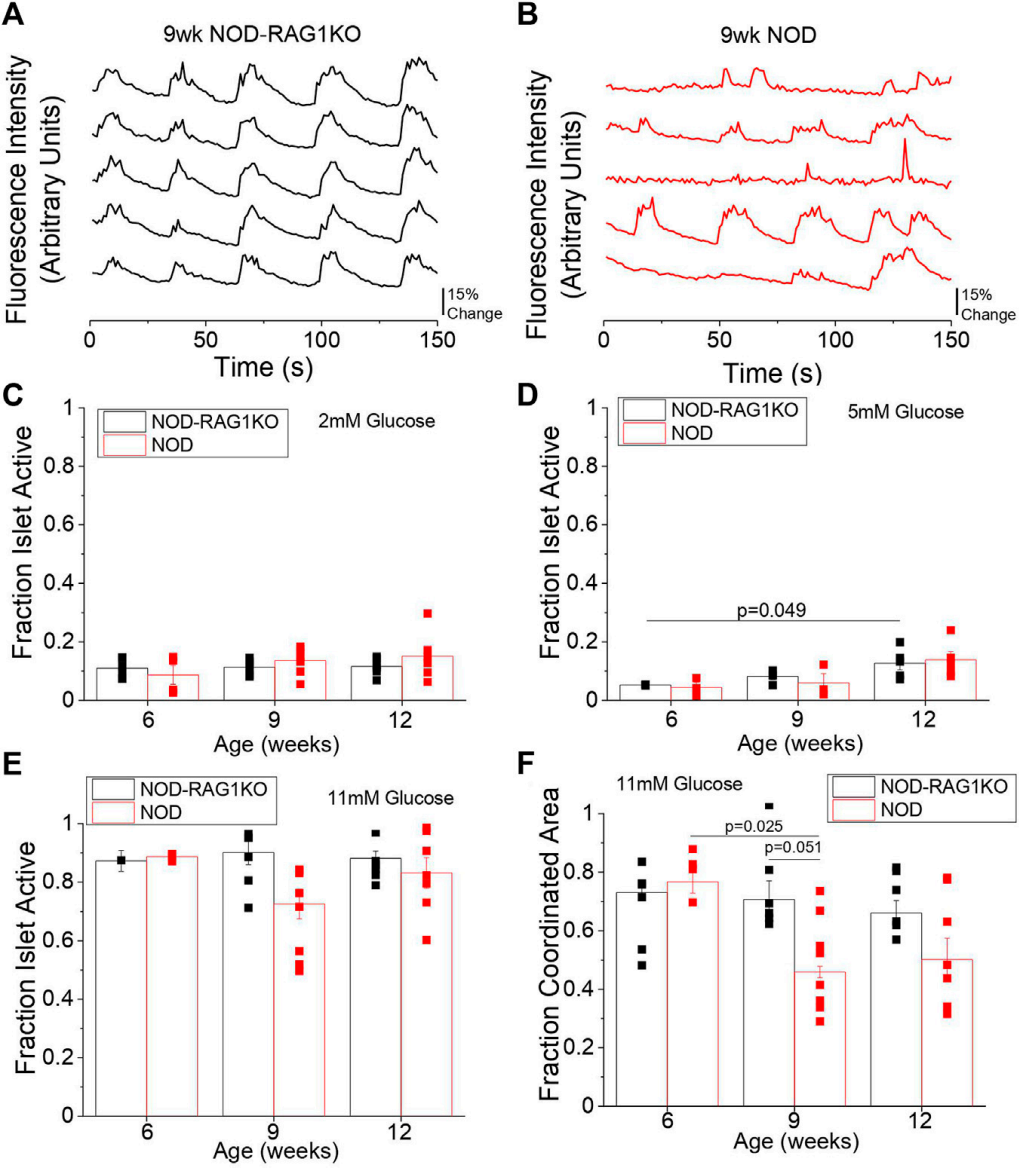
NOD mice have altered Ca^2+^ signaling as early as 9 weeks of age. Representative plots of intracellular Ca^2+^ as measured by fluorescence intensity at 11 mM glucose in 5 individual cells in the same islet over time in 9-week old **(A)** NOD-RAG1KO (black, *n* = 3–4) and **(B)** NOD (red, *n* = 3–5) mice. Quantification of the fraction of the islet area showing intracellular Ca^2+^ activity at **(C)** 2 mM glucose, **(D)** 5 mM glucose, and **(E)** 11 mM glucose in isolated islets from 6, 9, and 12-week old NOD and NOD-RAG1KO mice. **(F)** Quantification of the fraction of the islet area with coordinated Ca^2+^ oscillations at 11 mM glucose in isolated islets from 6, 9, and 12-week old NOD and NOD-RAG1KO mice. Data in C-F represents the mean ± SEM. Data from individual mice are represented by black and red squares for NOD-RAG1KO and NOD mice respectively (*n* = 3–6). *p* < 0.05 is significant as determined by ANOVA. All animals are female unless otherwise noted.

We next investigated changes in intracellular Ca^2+^ signaling dynamics and Cx36 gap junction coupling in islets from 6, 9, and 12-week old mice which were previously found to be normoglycemic. At 6 weeks no differences in Ca^2+^ signaling dynamics were observed between NOD and NOD-RAG1KO islets (Supplementary Figures S2A,B) at 11 mM glucose. At 9 and 12 weeks in the NOD mouse Ca^2+^ dynamics were visually disrupted (Figures 2A,B, Supplementary Figures S2C,D) with disrupted Ca^2+^ pulsatility and oscillation frequency compared to NOD-RAG1KO controls. We quantified the fraction of each islet that was active (i.e., pulsing intracellular Ca^2+^) and the fraction of the islet that showed coordinated Ca^2+^ oscillations. At 2, 11, and 20 mM glucose there were no significant differences in the fraction of the islet that was active between NOD and NOD-RAG1KO islets (Figures 2C,E, Supplementary Figure S2E) at any ages studied. At 5 mM glucose, there was no change in the fraction of the islet that was active in NOD islets compared with NOD-RAG1KO controls at any age studied (Figure 2D). At 11 mM glucose, there was a significant decrease in the fraction of the islet area with coordinated Ca^2+^ oscillations from 6 to 9 weeks in NOD mice (*p* = 0.025), compared to NOD-RAG1KO controls which were unchanged with age (Figure 2F). While the islet area with coordinated Ca^2+^ oscillations appears to decrease from 11 mM glucose to 20 mM glucose at 6 and 9 weeks of age (Figure 2F and Supplementary Figure S2F), this may be due to fast islet Ca^2+^ oscillations that could not be sampled at the 1 Hz imaging rate (Stozer, Klemen et al., 2021; Pohorec, Bombek et al., 2022). Overall, our results show that intracellular Ca^2+^ dynamics are disrupted in islets from NOD mice as early as 9 weeks of age.

### Islets From NOD Mice Show Reduced Connexin36 Gap Junction Coupling

**FIGURE 3:**
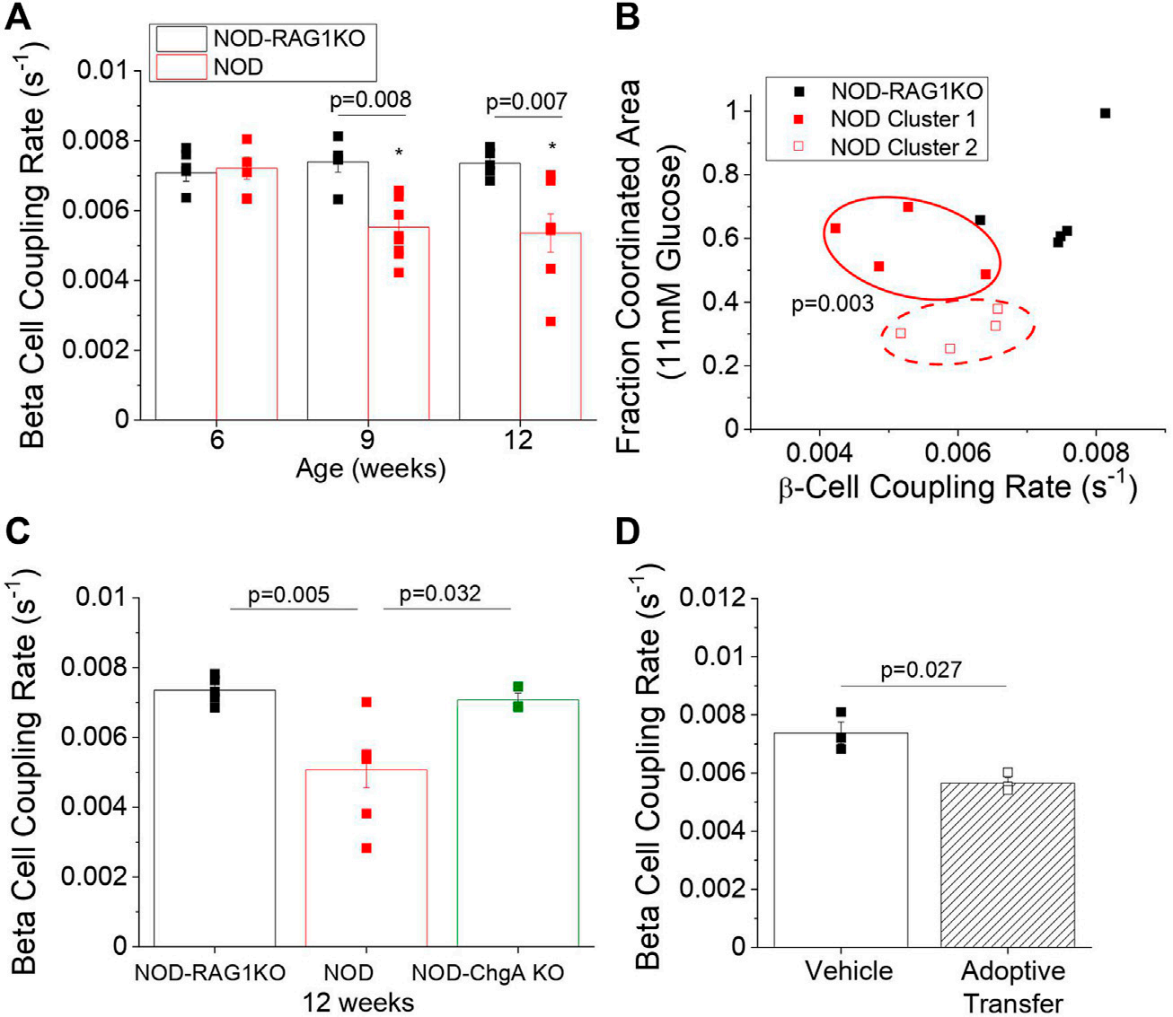
NOD mice have decreased β-cell coupling as early as 9 weeks of age and decreased β-cell coupling in the NOD mouse is mediated by autoreactive T-cells. **(A)** β-cell coupling as measured by fluorescence recovery rate in isolated islets from 6, 9, and 12-week old NOD (red, *n* = 6–9) and NOD-RAG1KO (black, *n* = 5–6) mice. Data represent the mean ± SEM. Data from individual mice are represented by black and red squares for NOD-RAG1KO and NOD mice respectively (*n* = 3–6). *p* < 0.05 is significant as determined by ANOVA. **(B)** K-means clustering analysis of the correlation between β-cell coupling and the fraction of the islet area with coordinated intracellular Ca^2+^ oscillations at 11 mM glucose in NOD and NOD-RAG1KO mice at 9 weeks of age. Red solid and dashed ovals indicate the clusters of animals identified by k-means analysis. **(C)** β-cell coupling as measured by fluorescence intensity recovery rate in isolated islets from 12-week old NOD (red), NOD-RAG1KO (black), and NOD chromogranin-A knockout (NOD-ChgA KO, green). Data from individual mice are represented by black, red, or green squares for NOD-RAG1KO, NOD, or NOD-ChgA KO mice respectively (*n* = 4–5). **(D)** β-cell coupling as measured by fluorescence intensity recovery rate in isolated islets from mice 2 weeks post-adoptive transfer with diabetogenic splenocytes or vehicle control. Data from individual mice are represented by solid or open squares for vehicle or adoptive transfer mice respectively (*n* = 3). Data in A and B represent the mean ± SEM. *p* < 0.05 is significant as determined by ANOVA in A and t-test in B. All animals are female unless otherwise noted.

To determine if islet gap junction coupling was also altered in NOD mice, we used FRAP to measure changes in β**-**cell Cx36 gap junction coupling. As early as 9 weeks of age, we observed a 25% decrease in β**-**cell coupling (*p* = 0.008) in NOD mice compared to NOD-RAG1KO controls (Figure 3A). This decrease in coupling persisted to 12 weeks in NOD mice (*p* = 0.007). We next determined if changes in β**-**cell coupling were predictive of a future diabetic phenotype. We used K-means clustering analysis of β**-**cell coupling from Figure 3A plotted against the fraction of the islet area with coordinated Ca^2+^ oscillations at 11 mM glucose from Figure 2F in 9-week old NOD and NOD-RAG1KO mice. We found that a subset of NOD mice with similar gap junction coupling and high Ca^2+^ coordination (solid red squares) clustered with the NOD-RAG1KO control mice (Figure 3B). A second subset of NOD mice with similar gap junction coupling and lower Ca^2+^ coordination (open red squares) clustered separate from both the NOD-RAG1KO islets and the NOD islets with higher coupling and coordination (Figure 3B). No significant correlation was found between gap junction coupling rate and Ca^2+^ coordination (Figure 3B). When comparing the coordinated area for each cluster of NOD mice, we found a significant (*p* = 0.003) difference in Ca^2+^ coordination between cluster 1 (mean = 0.58 ± 0.05 SEM) and cluster 2 (mean = 0.32 ± 0.03 SEM) identified using K-means clustering (Figure 3B). These results show that populations of NOD mice can be identified that have reduced gap junction coupling and disrupted Ca^2+^ dynamics.

### Decreased β-Cell Coupling in NOD-RAG1KO Mice Following Adoptive Transfer of Diabetic Splenocytes

We next determined if changes in β**-**cell gap junction coupling were mediated by activated immune cells, as changes in coupling occurred only in the NOD mice. To determine the role of activated T-cells in mediating disruptions to islet coupling, we utilized 12 week old NOD-RAG1KO mice, NOD mice, and NOD mice with a knockout of chromogranin A (NOD-ChgA KO). NOD-ChgA KO mice have a competent immune system, but do not develop T1D as they lack the primary antigen causing autoimmune diabetes in this strain of NOD mouse, ChromograninA (Baker, Bradley et al., 2016). NOD-ChgA KO mice showed significantly higher (*p* = 0.032) Cx36 gap junction coupling than age-matched NOD mice and showed similar levels of coupling as NOD-RAG1KO controls (Figure 3C). To further determine the role of diabetogenic T-cells in mediating decreases in β**-**cell coupling we transferred splenocytes from diabetic NOD mice into immunodeficient NOD-RAG1KO mice. Two weeks after splenocyte or vehicle transfer, islets from mice which received splenocytes had a 23% reduction (*p* = 0.027) in β**-**cell gap junction coupling compared to vehicle only control mice (Figure 3D). Overall, our results indicate that Cx36 coupling is reduced between β**-**cell by the presence of autoreactive T-cells.

### Modulation of Connexin36 Coupling and Intracellular Calcium Mediates Islet Survival With Pro-Inflammatory Cytokines

**FIGURE 4:**
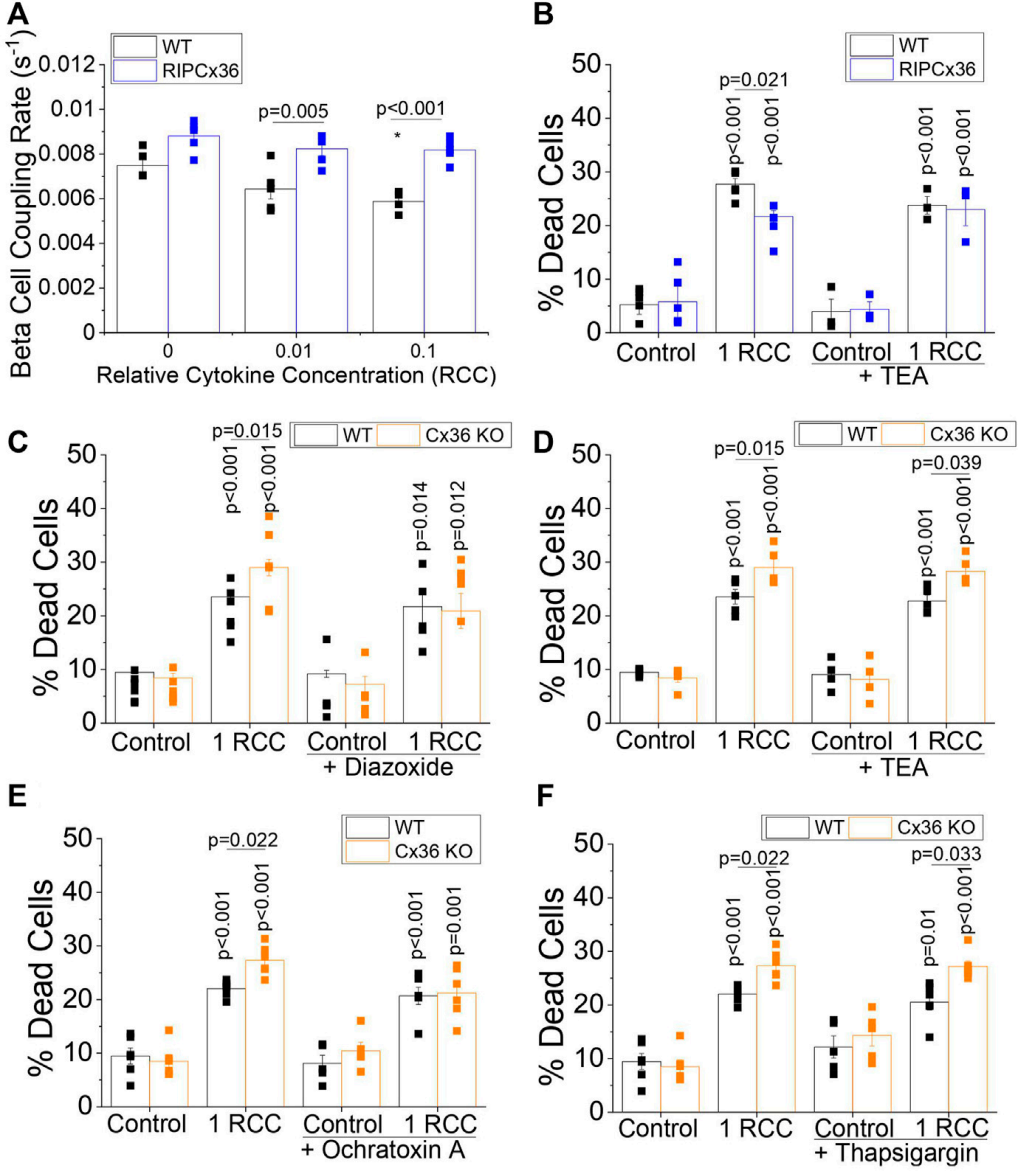
Modulation of Cx36 Coupling and Intracellular Ca^2+^ Mediate Cytokine-Induced Islet Death. **(A)** β-cell coupling rate as measured by FRAP in islets from WT and RIP Cx36 mice treated with 0, 0.01, or 0.1 relative cytokine concentration (RCC) for 24 h (*n* = 5). **(B)** Percent cell death islets from WT and RIP Cx36 mice treated with or without cytokines (1 RCC) and with or without the K^+^ channel inhibitor TEA for 24 h (*n* = 3–5). Percent cell death in islets from WT and Cx36 KO mice treated with or without **(C)** 250 nM of the K_ATP_ channel activator Diazoxide (*n* = 5) or **(D)** 20 mM of the K^+^ channel inhibitor TEA (*n* = 3) with or without a cytokine cocktail and for 24 h. Percent cell death in WT and Cx36 KO islets treated with or without **(E)** 1 µM of the SERCA activator Ochratoxin A (*n* = 5) or **(F)** 1 µM of the SERCA inhibitor thapsigargin (*n* = 5) with or without cytokines (1 RCC) for 24 h. Data represent the mean ± SEM. *p* < 0.05 is significant as determined by ANOVA. * Indicates *p* < 0.05 compared to untreated controls in A.

Increases in Cx36 coupling have been shown to protect against cytokine-induced death in the islet (Allagnat, Klee et al., 2013). Similarly, the Ca^2+^ channel blocker Verapamil has been shown to protect against β-cell death in patients with T1D, suggesting a role for intracellular Ca^2+^ in mediating islet survival (Ovalle, Grimes et al., 2018). As we have shown that both Cx36 and intracellular Ca^2+^ signaling are disrupted in islets from pre-diabetic NOD mice, we next wanted to test if modulating Cx36 coupling and intracellular Ca^2+^ (outlined in Supplementary Table S1) impacts islet survival in the context of pro-inflammatory cytokine treatment. In islets overexpressing Cx36 (RIP-Cx36), levels of β-cell coupling as measured by FRAP are maintained over increasing cytokine concentration, with 0.01 and 0.1 relative cytokine concentration (RCC), as compared to a x1 solution of 10 ng/ml TNF-α, 5 ng/ml IL-1β, and 100 ng/ml IFN-γ as described in the methods section. In contrast, WT controls showed decreased β-cell gap junction coupling with increasing cytokine concentration (Figure 4A). RIP Cx36 islets were significantly protected against cytokine-induced death (*p* = 0.021); however, increasing intracellular Ca^2+^ by closing K^+^ channels, (including K_ATP_ channels) with TEA abolished this protective effect (Figure 4B). In contrast, Cx36 KO islets showed significantly increased cytokine-induced death (*p* = 0.015) compared to WT controls, and this effect was completely abolished by decreasing intracellular Ca^2+^ by activating K_ATP_ channels with diazoxide (Figure 4C). This difference between WT or Cx36 KO islets was maintained upon increasing intracellular Ca^2+^ with TEA (Figure 4D). Thus modulating cytosolic Ca^2+^ improved cell survival in islets with a loss of Cx36 function compared to WT controls

We next tested the effects of modulating ER Ca^2+^ on the exacerbation of cell death in Cx36 KO islets by applying the SERCA channel activator ochratoxin A and inhibitor thapsigargin. Increasing ER Ca^2+^ with ochratoxin A reversed the elevation in cytokine-induced death in Cx36 KO islets (Figure 4E). In contrast upon decreasing ER Ca^2+^ cell death was still elevated in Cx36 KO islets (Figure 4F). While treatment with thapsigargin for 24 h did not significantly increase cell death in WT or KO islets, previous studies in mouse islet suggest that 48 h treatment may be required for significant levels of cell death in mouse islets (Duprez and Jonas 2011).

### Overexpression of Connexin36 Does Not Prevent Islet Dysfunction in the NOD Mouse

**FIGURE 5:**
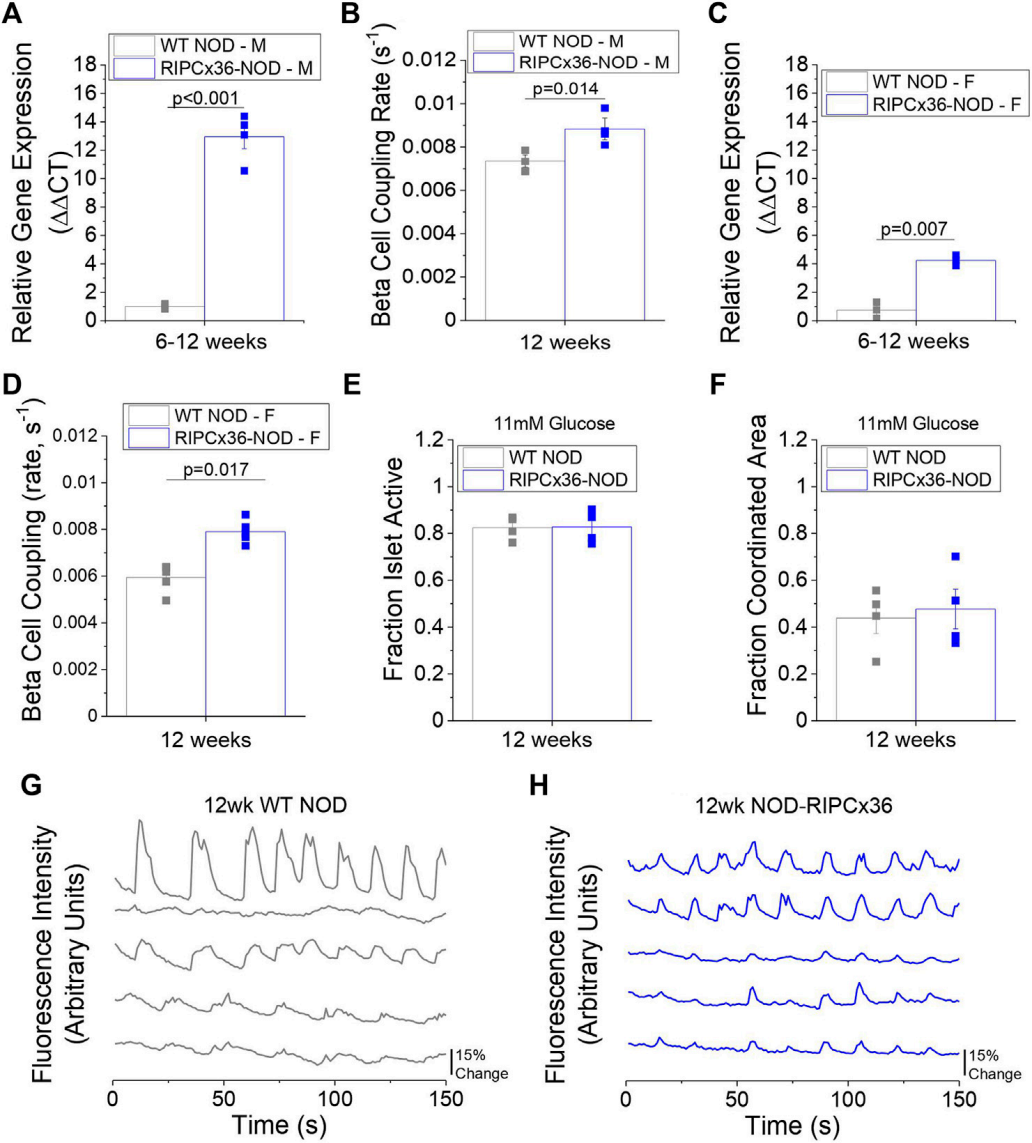
Overexpression of Cx36 in the NOD mouse. **(A)** Gene expression of Cx36 in 8–12-week old male WT NOD and RIPCx36-NOD mice normalized to HPRT1 housekeeping gene using the ΔΔCt method (*n* = 4). **(B)** β-cell coupling as measured by fluorescence recovery rate in isolated islets from 12-week old male WT NOD (grey) and RIPCx36-NOD (blue) mice (*n* = 4). **(C)** Gene expression of Cx36 in 12-week old female WT NOD and RIPCx36-NOD mice normalized to HPRT1 housekeeping gene using the ΔΔCt method (*n* = 3). **(D)** β-cell coupling as measured by fluorescence intensity recovery rate in isolated islets from WT NOD and RIPCx36-NOD mice (*n* = 4–5). Quantification of the fraction of the islet area showing intracellular Ca^2+^ activity **(E)** and the fraction of the islet area with coordinated Ca^2+^ oscillations **(F)** at 11 mM glucose in isolated islets from 12 week old WT NOD and RIPCx36-NOD mice (*n* = 3–4). Representative plots of intracellular Ca^2+^ as measured by fluorescence intensity at 11 mM glucose in 5 individual cells in the same islet over time in 12-week old **(G)** WT NOD (grey) and **(H)** RIPCx36-NOD (blue) mice. Data from individual mice are represented by grey or blue squares for WT NOD or RIPCx36-NOD mice respectively. Data in A-F represent the mean ± SEM. *p* < 0.05 is significant as determined by t-test in A-F. All animals are female unless otherwise noted.

Given that reduced Cx36 gap junction coupling can exacerbate cytokine-mediated β**-**cell death, we next examined the role of decreased Cx36 coupling in the pathogenesis of diabetes in the NOD mouse. To accomplish this, we backcrossed a β**-**cell specific Cx36 overexpression onto the NOD background (Klee, Allagnat et al., 2011). In 8–12-week old male NOD mice overexpressing Cx36 (RIPCx36-NOD) islets had ∼x13 higher Cx36 gene expression (*p* < 0.001, Figure 5A) and had 20% increased gap junction coupling compared to WT NOD islets (*p* = 0.014, Figure 5B). Islets from 12-week old female RIPCx36-NOD mice had ∼x6 higher Cx36 gene expression (*p* = 0.007, Figure 5C) and had ∼x1.3 higher gap junction coupling (*p* = 0.017, Figure 5D) compared to WT NOD controls. Of note, WT NOD islets had similar levels of Cx36 gap junction coupling compared to islets from the original NOD colony. These results confirm the successful development of a mouse model with increased Cx36 expression and gap junction coupling.

To determine if increasing Cx36 coupling improved islet function in islets from NOD mice, we analyzed intracellular Ca^2+^ signaling dynamics and *in vitro* insulin secretion. No differences in the fraction of the islet active was observed at any glucose concentration studied in RIPCx36-NOD islets compared to WT NOD islets (Figure 5E, Supplementary Figures S3A,B). Similarly, while RIPCx36-NOD islets showed a trend to slightly higher coordinated Ca^2+^ oscillations, this was not significantly different compared to WT NOD islets (Figure 5F). Overall, intracellular Ca^2+^ dynamics at 11 mM glucose were similar in islets from 12-week old RIPCx36-NOD mice compared to WT NOD mice (Figures 5G,H). Insulin secretion at both 2 mM glucose and 20 mM glucose was similar in both WT NOD and RIPCx36-NOD islets (Supplementary Figure S3C). Overall, our data indicate that increased Cx36 coupling has little effect on islet function as measured by Ca^2+^ dynamics or *in vitro* insulin secretion.

### Increased Connexin36 Coupling Protects Against Early Diabetes Onset in the NOD Mouse

**FIGURE 6:**
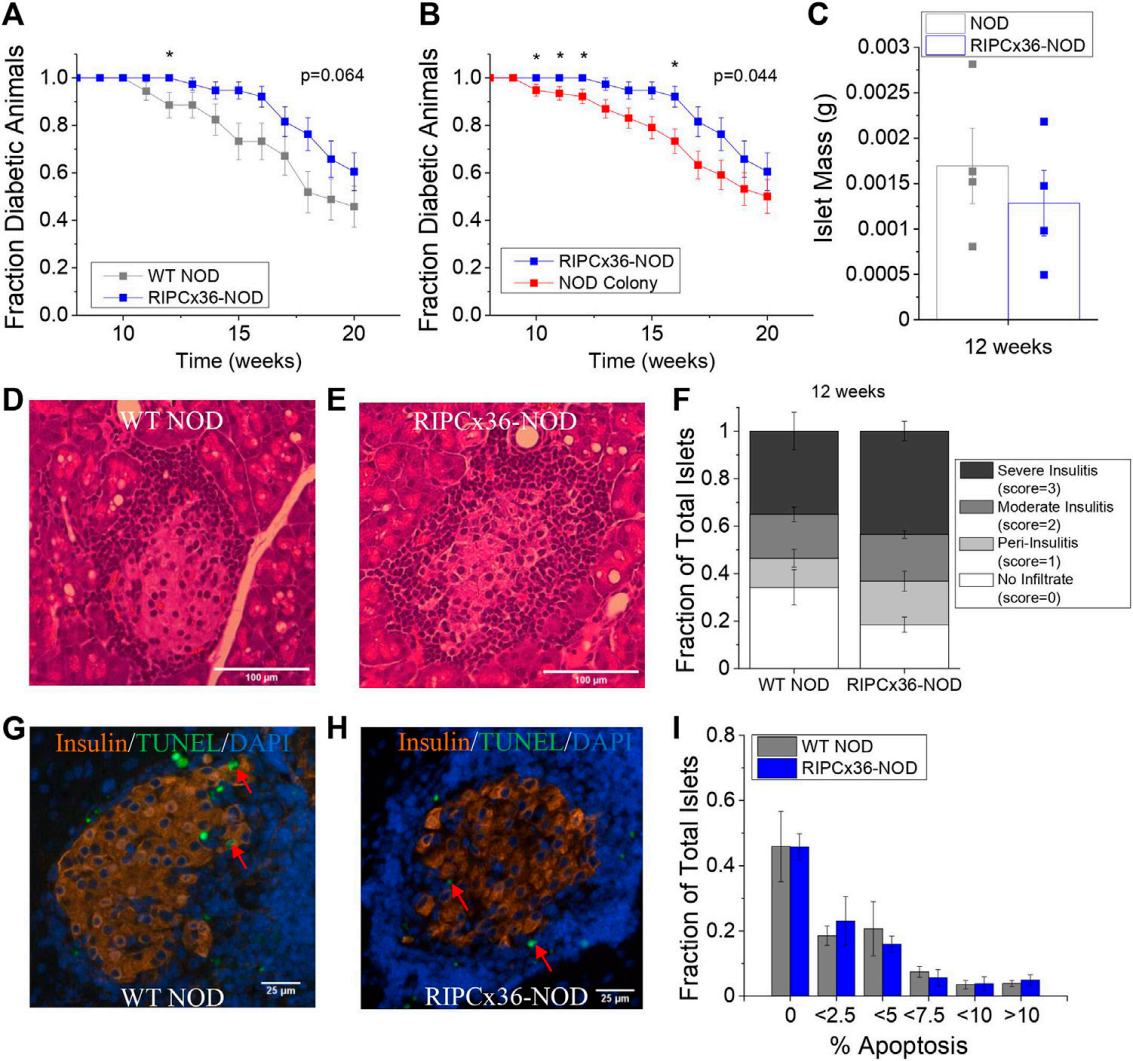
Overexpression of Cx36 protects against T1D onset between 10–16 weeks of age in the NOD mouse. **(A)** Kaplan-Meier survival analysis of diabetes onset in WT NOD (grey, *n* = 34) and RIPCx36-NOD (blue, *n* = 38) mice. *p* < 0.05 indicates a significant difference in survival probability as determined by the Breslow test. **(B)** Kaplan-Meier survival analysis of diabetes onset in NOD mice (NOD colony, red, *n* = 77) and RIPCx36-NOD (blue, *n* = 38) mice. *p* < 0.05 indicates a significant difference in survival probability as determined by the Breslow test. **(C)** Average islet mass per pancreas in 12-week old WT NOD (grey) and RIPCx36-NOD (blue) pancreata. Representative images of H&E staining in WT NOD **(D)** insulitis score = 2 and RIPCx36-NOD **(E)** insulitis score = 3 pancreas. **(F)** Fraction of total islets averaged over 6 slices per pancreas with an insulitis score of 0, 1, 2, or 3 in 12-week old WT NOD (*n* = 5) and RIPCx36-NOD (*n* = 6) mice as determined by IHC. Representative images of TUNEL and insulin staining in pancreas sections from WT NOD **(G)** and RIPCx36-NOD **(H)** mice. Red arrows indicate TUNEL+ insulin + cells. **(I)** Histogram of the average fraction of total islets with increasing percentage of apoptotic (TUNEL^+^) insulin^+^ cells per islet in WT NOD (grey, *n* = 4) and RIPCx36-NOD (blue, *n* = 4) islets. Data represents the mean ± SEM. All animals are female unless otherwise noted.

While overexpression of Cx36 did not significantly protect against islet dysfunction, we next determined if increased Cx36 coupling protected against β**-**cell death and diabetes development. Female RIPCx36-NOD mice were significantly protected against developing diabetes at 12 weeks as determined by 95% confidence intervals compared to WT NOD controls (Figure 6A); however, the long-term survival of the RIPCx36-NOD mice as determined by Kaplan-Meier survival analysis was not significantly different than the WT NOD (*p* = 0.064, Figure 6A). Compared to the female mice in the original NOD colony, the female RIPCx36-NOD mice were significantly protected against diabetes onset as determined by 95% confidence intervals at 10, 11, 12, and 16 weeks (Figure 6B). The survival of the RIPCx36-NOD mice was also significantly higher than that of the NOD colony as determined by Kaplan-Meier survival analysis (*p* = 0.044, Figure 6B). There was no significant difference in survival between the WT NOD and NOD colony female mice (Supplementary Figure S3D). While the average age of diabetes onset decreased with each subsequent generation, there was no significant difference in the overall average age of diabetes onset between the WT NOD and original NOD colony (Supplementary Figure S3E).

We next quantified cell death and immune cell infiltration in pancreas sections from WT NOD and RIPCx36-NOD female mice at 12 weeks where significant protection was observed (Figures 6D,E). No significant differences in immune infiltration (insulitis) or total islet mass as measured by insulin staining (Supplementary Figure S4A) were observed between pancreata from WT NOD and RIPCx36-NOD mice (Figure 6F). Similarly, we observed no difference in the percentage of apoptotic cells per islet as determine by TUNEL staining (Figures 6G,H, Supplementary Figure S4B) or the distribution of β**-**cell death per islet in islets from WT NOD and RIPCx36-NOD mice (Figure 6I). Overall, our data indicate that increased Cx36 coupling has a modest impact in protecting against diabetes progression but without detctable changes in insulitis or β**-**cell death.

## Discussion

The goal of this study was to determine the role of altered Cx36 coupling and Ca^2+^ signaling in the development of T1D in the NOD mouse. We found that euglycemic NOD mice had disrupted Ca^2+^ signaling dynamics and decreased Cx36-mediated gap junction coupling at 9 and 12 weeks age, compared to age-matched immunodeficient controls. These changes occurred prior to marked dysglycemia. Decreases in Cx36 gap junction coupling were mediated by autoreactive T-cells. Increases in Cx36 gap junction coupling and pharmacological agents that decreased cytosolic (Ca^2+^) and increased ER Ca^2+^ improved the survival of cytokine treated islets *in vitro*. While overexpression of Cx36 in the β-cells of NOD mice protected against early diabetes onset (at ∼12 weeks), increased Cx36 gap junction coupling did not significantly improve islet function or survival, resulting in only modest protection against T1D development. Our results suggest that while decreases in β-cell Cx36 gap junction coupling and altered Ca^2+^ signaling dynamics are associated with the development of T1D in the NOD mouse, increasing gap junction coupling alone is not sufficient to prevent the onset of disease.

### Connexin36 Coupling is Decreased in Islets From NOD Mice and Correlates With Islet Dysfunction

Consistent with studies in prediabetic mouse models of type 2 diabetes (Carvalho, Oliviera et al., 2012; Gonzalez, Merino et al., 2013; Irles, Neco et al., 2015; Amaral, Kravets et al., 2020), we found that Cx36 gap junction coupling is decreased in islets from prediabetic NOD mice as early as 9 weeks of age compared to immunodeficient controls. Several studies have shown that glucose-induced Ca^2+^ oscillation coordination is mediated by electrical coupling between β**-**cells that coordinates membrane depolarization and subsequent Ca^2+^ influx across the islet (Ravier, Guldenagel et al., 2005; Benninger, Zhang et al., 2008; Perez-Armendariz 2013). This is supported by our data, where decreases in the coordination of glucose-induced Ca^2+^ oscillations directly correlated with decreases in Cx36 coupling. While we observed dysfunction to Cx36 coupling and Ca^2+^ oscillations in islets from NOD mice as early as 9 weeks of age, we did not observe dysfunction to insulin secretion at any of the time points studied. This is in line with previous studies where total KO of Cx36 in the islet disrupts insulin release pulsatility, but has a limited effect on overall insulin secretion levels (Head, Orseth et al., 2012). Our previous study has shown low levels of pro-inflammatory cytokines decrease Cx36 coupling and coordination of glucose-stimulated Ca^2+^ oscillations without causing dysfunction to levels of insulin secretion (Farnsworth, Walter et al., 2016). While decreased Cx36 coupling may lead to a shift in the range of glucose responsiveness observed in this study and our previous study, measurement of GSIS in isolated islets does not provide sufficient temporal resolution to determine shifts in glucose responsiveness and future study is required to determine any potential contributions to insulin secretion with loss of Cx36 coupling. In the early stages of T1D, levels of pro-inflammatory cytokines may be low enough to cause dysfunction to Cx36 gap junction coupling and Ca^2+^ dynamics with no significant effects on insulin secretion, as shown in this study. This is also supported by findings in type 2 diabetes, where low-grade inflammation caused impaired Ca^2+^ handling and islet dysfunction (Dula, Jecmenica et al., 2010). While measuring changes in Ca2+ dynamics in advanced stages of T1D in isolated mouse islets is technically challenging, future studies utilizing NOD pancreas slices from animals with advanced disease, coupled with immunocytochemical labeling of β-cells and T-cells, couldp provide insight into further alterations in Cx36 coupling and Ca^2+^ dynamics in T1D progression. Altogether, our results indicate a role for decreased Cx36 coupling in mediating islet dysfunction in early type 1 diabetes.

We also identified sub-populations of NOD mice with higher levels of dysfunction as determined by the decreased coordination of glucose-stimulated Ca^2+^ oscillations. This heterogeneity in islet function was not present in the immunodeficient control mice and therefore is likely associated with diabetes pathogenesis. However, we were unable to follow these mice to determine if this increased dysfunction correlated with earlier development of diabetes. We also noted that there was no correlation between gap junction coupling and Ca^2+^ oscillation coordination in islet from NOD mice, suggesting that changes in gap junction coupling alone are not representative of islet dysfunction. The identified sub-population of NOD mice with lower Ca^2+^ oscillation coordination did not have significantly different levels of coupling compared to the population of NOD mice with increased Ca^2+^ oscillation coordination. This suggests that other factors, such as K_ATP_ channel function or ER/mitochondrial Ca^2+^ buffering may also be impacted in diabetic animals; however, further study is required to determine how Ca^2+^ handling is altered. Previous studies in twins who were at risk for developing T1D have shown a positive correlation between decreases in first-phase insulin release and onset of T1D, supporting our hypothesis that greater islet dysfunction prior to changes in glucose homeostasis is associated with disease progression (Lo, Hawa et al., 1992). As altered first-phase insulin secretion occurs with a loss of Cx36 gap unction coupling in the islet, correlations between first-phase insulin secretion with levels of Cx36 coupling and altered Ca^2+^ signaling may predict disease onset.

### Decreased Connexin36 Coupling is Mediated by Activated Immune Cells From the NOD Mouse

Our results indicate that decreases in Cx36 coupling occur as a result of T1D onset, as levels of Cx36 are similar in NOD mice and immunodeficient controls at early ages but decrease starting at 9 weeks in the NOD mice. Deletion of chromogranin A in NOD mice, which lack autoreactive T-cells, preserves Cx36 coupling and transfer of diabetogenic splenocytes to immunodeficient mice decreases Cx36 coupling in islets (Baker, Bradley et al., 2015). Thus autoreactive immune cells are both sufficient and required to cause decreases in Cx36 gap junction coupling in the NOD mouse, which has not been previously shown. Islet dysfunction, specifically changes in islet electrophysiology, prior to diabetes onset has not been well characterized in T1D. This is partly due to the heterogeneity of T1D onset making it difficult to pinpoint the time when decreased islet function can be observed in the absence of significant cell death (Matthews, Xue et al., 2015). Additionally, techniques used to measure changes in islet electrophysiology frequently require destruction of the 3D islet architecture. Utilizing gap junction coupling and changes in Ca^2+^ dynamics as a measure of islet function and changes in electrophysiology provides an alternative technique to capture early decreases in function of islet in pre-diabetic NOD mice. Our results are in line with previous studies that showed islet dysfunction as measured by a reduction in first-phase insulin secretion in at-risk children who went on to develop T1D (Sherry, Tsai et al., 2005). Activated immune cells likely decrease Cx36 gap junction coupling by production of high levels of pro-inflammatory cytokines, which we have shown decrease Cx36 gap junction coupling and alter Ca^2+^ dynamics *in vitro via* activation of protein kinase C δ (Farnsworth, Walter et al., 2016).

### Modulation of Intracellular and Endoplasmic Reticulum Calcium Compensates for Loss of Connexin36 Coupling in Cytokine Treated Islets

Our results indicate that modulation of Cx36 gap junction coupling in the islet can mediate the effects of pro-inflammatory cytokines on islet survival. Gap junction coupling and Ca^2+^ regulation are tightly coupled, suppressing spontaneous elevations at low glucose levels and coordinating pulsatile Ca^2+^ at high glucose. Elevations in Ca^2+^ can also exacerbate apoptosis, thus suggesting that Cx36 gap junction coupling protects against cell death *via* Ca^2+^ regulation (Nicotera and Orrenius 1998). Both diazoxide activation of K_ATP_ channels, and OTA-activation of SERCA which will suppresses cytosolic Ca^2+^ elevations, counteracted the impact of a loss of Cx36 on cell death. The protection against cell death afforded by Cx36 overexpression was eliminated upon TEA inhibition of K^+^ channels, which will elevate cytosolic Ca^2+^ elevations. Together these findings indicate that a loss of Cx36 gap junction coupling exacerbates cytokine-mediated cell death *via* elevated spontaneous Ca^2+^ activity. This is supported by previous studies, where loss of gap junction coupling in islet β-cells resulted in increased intracellular Ca^2+^ at low glucose levels, likely due to heterogeneity in β-cell excitability in the islet (Benninger, Head et al., 2011; Benninger, Dorrell et al., 2018). This conclusion is supported by studies where cytokines cause apoptosis in β-cells by increasing intracellular Ca^2+^ (Chang, Cho et al., 2004). Additionally, reduced ER Ca^2+^ has been linked to ER stress induced apoptosis in β-cells, suggesting that increasing ER Ca^2+^ with the SERCA activator may protect against cell death (O'Neill, Lu et al., 2013). Further studies are required to determine if increasing ER Ca^2+^ can protect against cytokine-mediated cell death by reducing ER stress in islets with decreases gap junction coupling. Overall, our results support a role for elevated intracellular Ca^2+^ as a result of a loss of Cx36 coupling that exacerbates cytokine-induced islet cell death. However, the exact mechanisms by which Cx36-regulated Ca^2+^ impacts Cx36-mediated protection require further studies.

### Increased β-Cell Connexin36 Coupling in the NOD Mouse Does Not Protect Against Islet Dysfunction or β-Cell Death

Our results indicate that decreases in Cx36 coupling are a result of T1D pathogenesis, therefore we also sought to determine if increasing Cx36 coupling could protect against the onset of disease in the NOD mouse. Overexpression of Cx36 did increase coupling at all time points studied; however, protection against disease onset was only observed early on from 10 to 12 weeks with a small difference in overall survival. We did not observe a significant improvement in islet Ca^2+^ dynamics or insulin secretion. These results are in contrast to our *in vitro* studies, as well as a previous study that used glibenclamide to increase Cx36 gap junction coupling and observed protection against T1D onset in the NOD mouse (Lamprianou, Gysemans et al., 2016). Glibenclamide also increases glucose-stimulated insulin secretion *via* K_ATP_ channel closure (Charpentier, Cancela et al., 2007; Ashcroft and Rorsman 2013), which may explain why improved insulin secretion was observed. While we found that overexpression of Cx36 increases β**-**cell gap junction coupling, dysfunction to intracellular Ca^2+^ dynamics remained. This suggests that glucose-stimulated Ca^2+^ signaling can be disrupted by changes in ER Ca^2+^ uptake in addition to decreased Cx36 coupling, as has been suggested by previous studies *in vitro* (Ramadan, Steiner et al., 2011; Kono, Ahn et al., 2012), and that increases in Cx36 gap junction coupling alone are insufficient to overcome altered intracellular Ca^2+^ handling.

Several studies have shown that Cx36 gap junction coupling between β-cells protects against cytokine-induced β**-**cell death *in vitro* (Klee, Allagnat et al., 2011; Allagnat, Klee et al., 2013) as well as streptozotocin-induced β-cell death *in vivo* (Klee, Allagnat et al., 2011). In contrast, we observed that increased Cx36 gap junction coupling provided little protection against β-cell apoptosis in NOD mice at 12 weeks, where mice were normoglycemic but significant insulitis was observed. This could be a result of the additional disruption to Ca^2+^ handling which was not recovered by increased Cx36 gap junction coupling. For example, recovery of ER Ca^2+^ homeostasis can protect against cytokine-mediated β-cell death (Clark, Kanekura et al., 2017), which follows with our results *in vitro* with K_ATP_ and SERCA modulators. The lack of protection against β-cell death with increased Cx36 gap junction coupling may also be due to β-cell death mediated by Ca^2+^-independent activation of the Fas ligand on the β-cell surface by activated immune cells (Lightfoot, Chen et al., 2012), as we did not observe differences in insulitis between NOD mice with normal or increased Cx36 gap junction coupling. While increases in Cx36 gap junction coupling alone is insufficient to protect against immune-mediated β-cell death in the NOD mouse, combination therapies which increase Cx36 gap junction coupling and recover ER Ca^2+^ homeostasis may show greater protection against β**-**cell death in T1D. Future studies that investigate changes in cytosolic and ER Ca^2+^ using genetically encoded and organelle targeted fluorescent Ca^2+^ probes may help to determine if modulating both intracellular Ca^2+^ and gap junction coupling can maintain ER Ca^2+^ homeostasis and normalize β-cell electrophysiology in islets from diabetic NOD mice.

## Conclusion

In summary, we have shown that Cx36 gap junction coupling is reduced in the islets of NOD mice prior to changes in glucose homeostasis. This decrease in Cx36 gap junction coupling is accompanied by altered coordination of Ca^2+^ dynamics across the islet and is mediated by autoreactive immune cells in the NOD mouse. *In vitro*, loss of Cx36 gap junction coupling exacerbates cell death and Cx36 overexpression protects against cell death *via* regulating cytosolic Ca^2+^. While increasing Cx36 gap junction coupling in the NOD mouse protected against early onset of T1D; long term survival, islet function, and β-cell death were not improved. Thus while islet dysfunction occurs prior to changes in glucose homeostasis in T1D, increased β-cell gap junction coupling alone is insufficient to recover islet function or prevent diabetes onset. Future studies will determine if increasing Cx36 coupling in combination with therapies which restore Ca^2+^ homeostasis can protect against islet dysfunction and death in the NOD mouse.

## Data Availability

The raw data supporting the conclusions of this article will be made available by the authors, without undue reservation.

## Nomenclature

AUC: Area under the curve
ANOVA: Analysis of variance
Ca^2+^: Calcium
C_t_: Cycle time
ChGAKO-NOD: Chromogranin A knockout NOD mouse
Cx36: Connexin36
FRAP: Fluorescence recovery after photobleaching
HBSS: Hanks balanced salt solution
HPRT1: hypoxanthine phosphoribosyltransferase 1
IFN-γ: Interferon gamma
IL-1β: Interleukin-1beta
RIP: Rat insulin promoter
RCC: Relative cytokine concentration
TNF-α: Tumor necrosis factor alpha
WT: Wild type
