# Supplementary material for: Modulation of Gap Junction Coupling Within the Islet of Langerhans During the Development of Type 1 Diabetes
Source: Front Physiol. 2022 Jun 28;13:913611. doi: 10.3389/fphys.2022.913611 (PMC9274093; doi:10.3389/fphys.2022.913611)
Supplement: Supplementary file 1 [file DataSheet1.PDF]

Supplementary Material

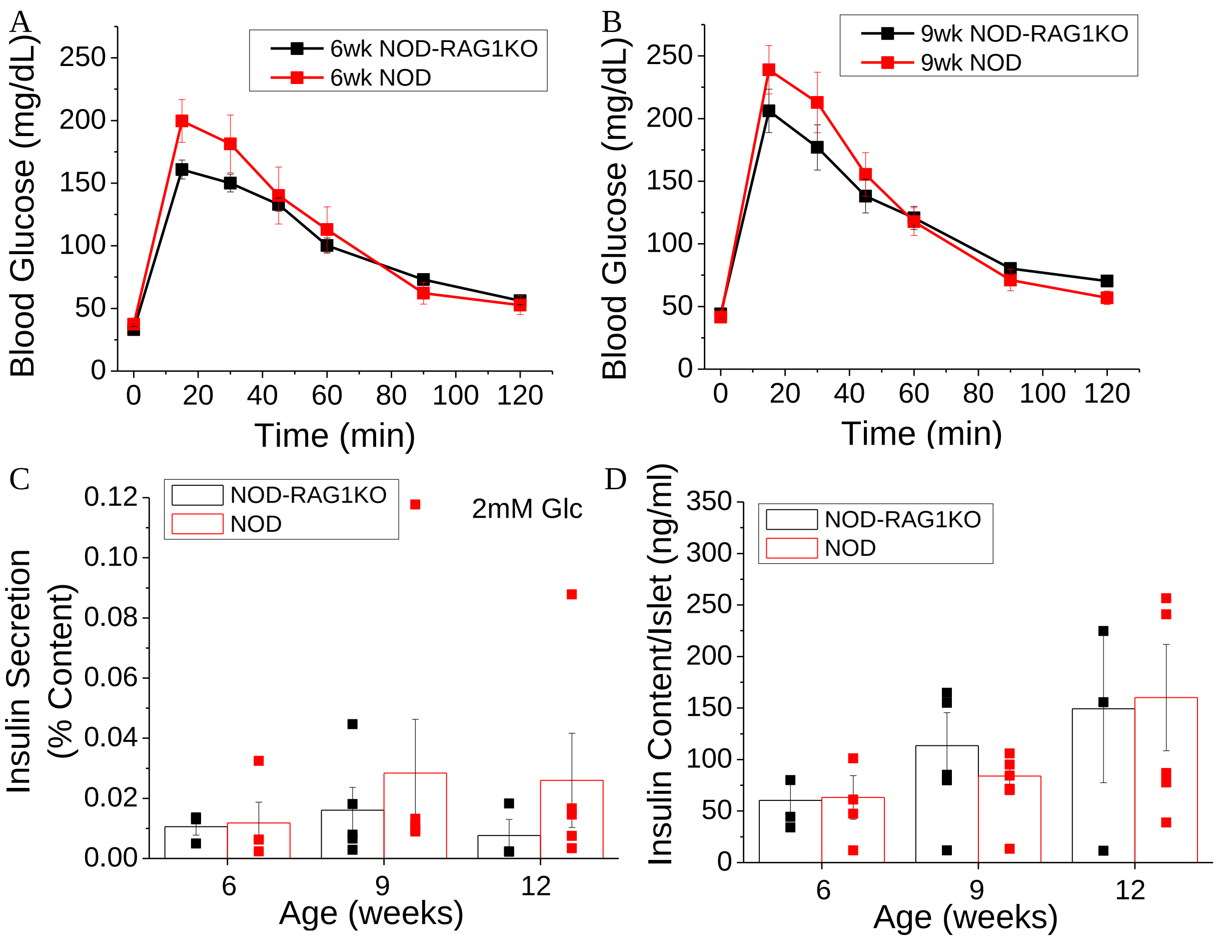

Supplemental Figure 1: Blood glucose and insulin secretion in NOD and NOD-RAG1KO mice. Blood glucose levels in (A) 6-week old and (B) 9 week old NOD (red) and NOD-RAG1KO (black) mice following a glucose tolerance test starting at time 0. (C) Insulin secretion normalized to insulin content in isolated islets from 6, 9, and 12-week old NOD and NOD-RAG1KO mice at 2mM glucose. (D) Insulin content per islet in isolated islets from NOD and NOD-RAG1KO mice as in D. Data in all panels represents the mean ± SEM. In C-E, data from individual mice are represented by black and red squares for NOD-RAG1KO and NOD mice respectively.

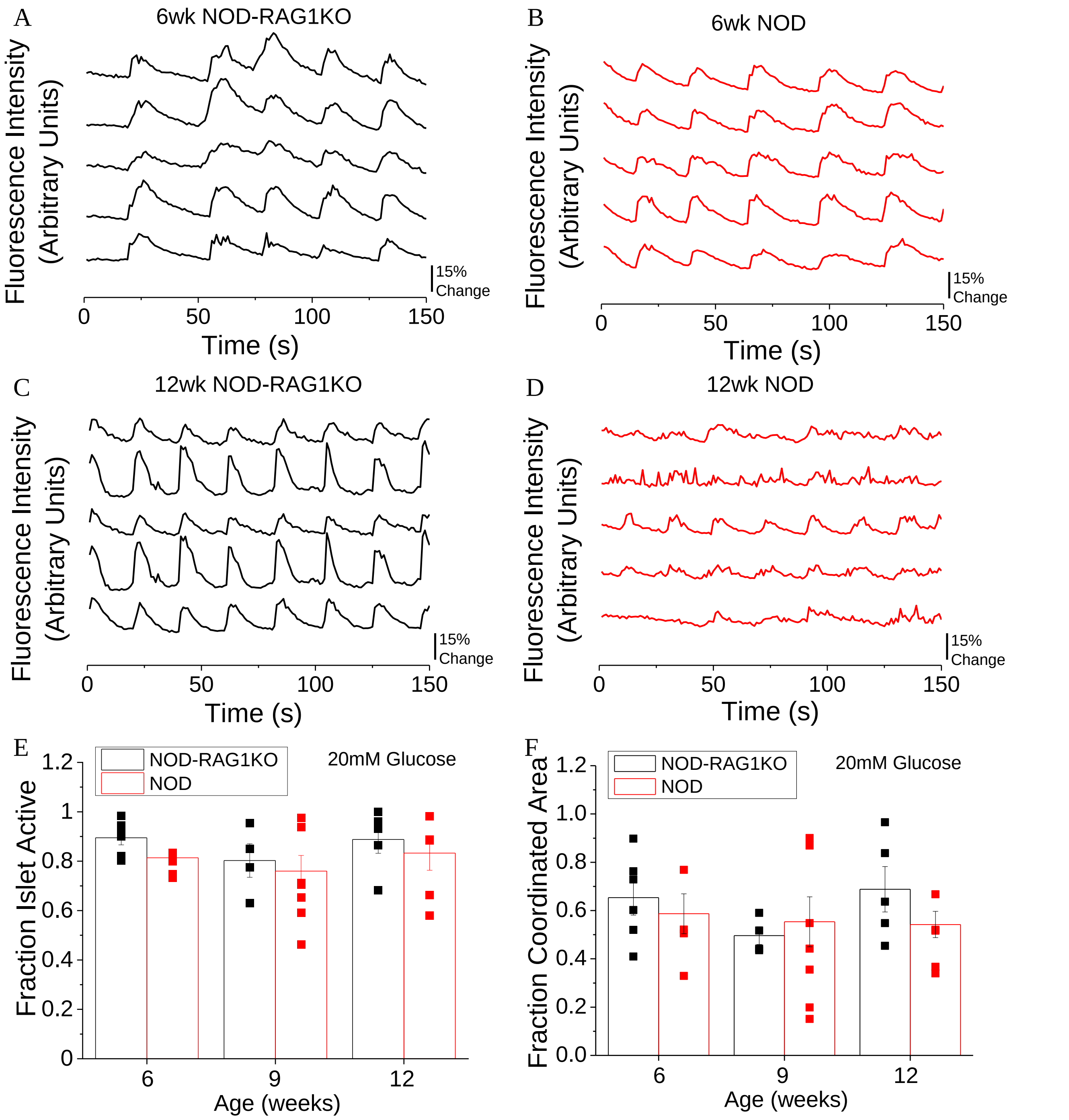

Supplemental Figure 2: Intracellular Ca^{2+} signaling in NOD and NOD-RAG1KO mice. Representative plots of intracellular Ca^{2+} as measured by fluorescence intensity at 11mM glucose in 5 individual cells in the same islet over time in 6 week NOD-RAG1KO (black) (A), 6 week NOD (red) (B), 12 week NOD-RAG1KO (C), and 12 week NOD mice (D). Quantification of (E) the fraction of the islet area showing intracellular Ca^{2+} activity and (F) the fraction of the islet area with coordinated Ca^{2+} oscillations at 20mM glucose in isolated islets from 6, 9, and 12 week old NOD and NOD-RAG1KO mice. Data in E and F represents the mean ± SEM. Data from individual mice are represented by black and red squares for NOD-RAG1KO and NOD mice respectively (n=3-6).

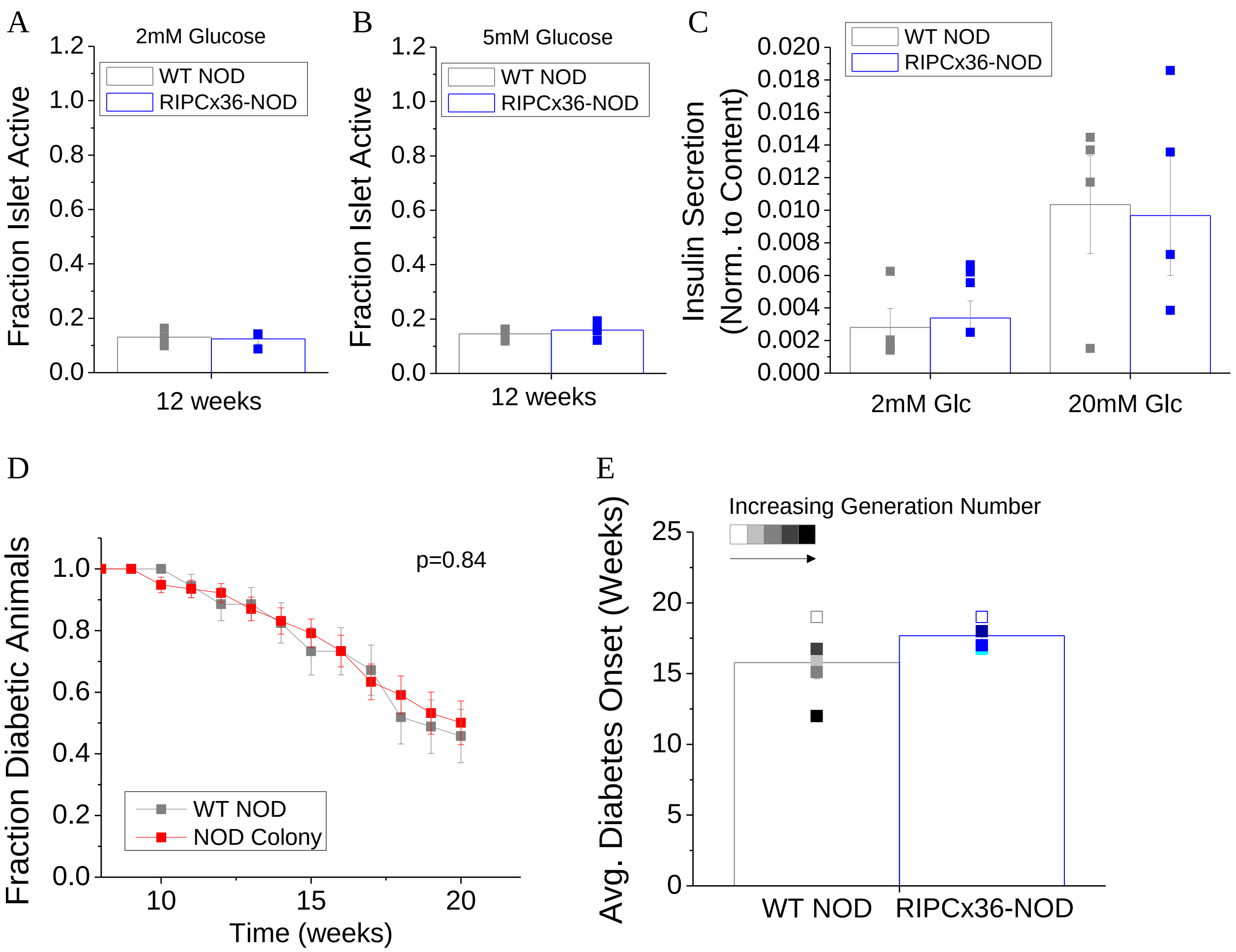

Supplemental Figure 3: Characterization of WT NOD and RIPCx36-NOD diabetic phenotype. Quantification of the fraction of the islet area showing intracellular Ca^{2+} activity at (A) 2mM glucose and (B) 5mM glucose in isolated islets from 12-week old WT NOD and RIPCx36-NOD mice (n=3-4). (C) Insulin secretion in isolated islets from 12-week old WT NOD and RIPCx36-NOD mice at 2 and 20mM glucose (n=4). (D) Kaplan-Meier survival curves for diabetes incidence in WT NOD (grey) and NOD colony (red) mice with Breslow test for significance between survival distributions, where $p<0.05$ is significant. (E) Average age of diabetes onset in WT NOD (grey) and RIPCx36-NOD (blue) mice. The average age of diabetes onset for each generation of mice is represented by black and blue squares for WT NOD and RIPCx36-NOD mice respectively, where increasing color intensity of the squares indicates increasing generation number (n=4-5). Data in all panels represents the mean ± SEM. Data from individual mice are represented by black, red, grey, or blue squares for NOD-RAG1KO, NOD, WT NOD, and RIPCx36-NOD mice respectively. $p<0.05$ is significant as determined by ANOVA.

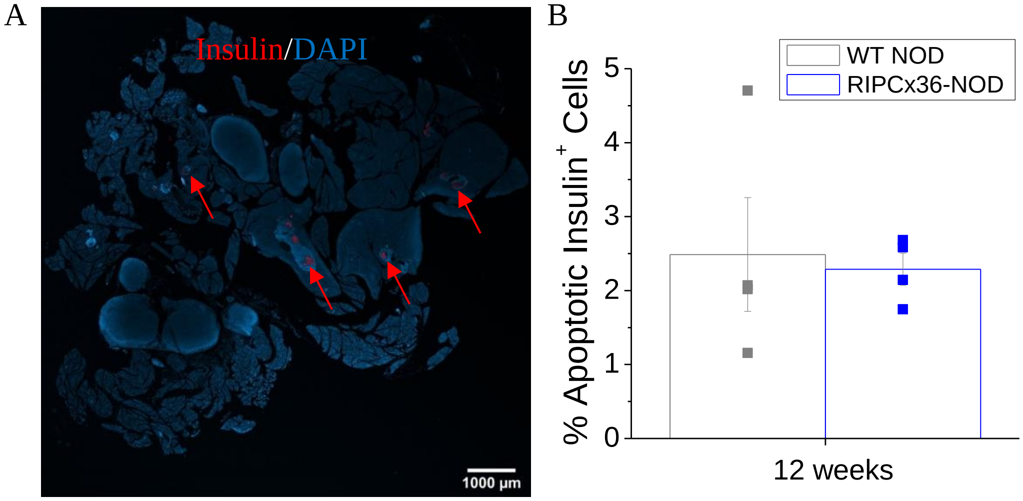

Supplemental Figure 4: Characterization of WT NOD and RIPCx36-NOD Islet Mass and Beta Cell Death. (A) Representative image of a pancreas slice from an NOD mouse stained using IHC for insulin (orange) and nuclei (DAPI, blue) where individual images were stitched together to achieve a continuous image of the entire pancreas slice. Pancreatic islets are indicated by red arrows. (B) Percent apoptotic (TUNEL$^+$) insulin$^+$ cells per islet averaged over 6 slices per pancreas in 12-week old WT NOD (grey) and RIPCx36-NOD (blue) pancreata. Data represents the mean ± SEM. Data from individual mice are represented by grey or blue squares for WT NOD and RIPCx36-NOD mice respectively.

Agent	Pharmacological Role	Anticipated Ca^{2+} Modulation
TEA	Closes K_{ATP} channels	Increased cytosolic Ca^{2+}
Diazoxide	Opens K_{ATP} channels	Decreased cytosolic Ca^{2+}
Ochratoxin A	Open SERCA channels	Increased ER Ca^{2+}, decreased cytosolic Ca^{2+}
Thapsigargin	Closes SERCA channels	Decreased ER Ca^{2+}, increased cytosolic Ca^{2+}

Supplementary Table 1: Outline of therapeutic agents cultured with isolated islets, the expected pharmacological role in the cell, and the anticipated modulation of intracellular Ca^{2+} with treatment.
